# Supplementary material for: Exploring pathogenesis and biomarkers through establishment of a rat model of male infertility with liver depression and kidney deficiency
Source: PLoS One. 2024 May 20;19(5):e0303189. doi: 10.1371/journal.pone.0303189 (PMC11104592; doi:10.1371/journal.pone.0303189)
Supplement: Attachment — Submitted filename: renamed_07136.docx [file pone.0303189.s005.docx]

The authors employed CRS in conjunction with adenine, trying to establish a clinically representative rat model which they named LD & KD in MI. Also, utilizing metabolomics techniques in tandem with bioinformatics analysis, the most significant metabolic pathways, biomarkers, and targets were identified. Overall, this work is a relatively novel study of the male infertility. A few questions and suggestions merit further investigation in this work.

1. I do not know whether the term LD & KD in MI claimed by the author accurately describes the disease state of the animal model. I am confused about whether the disease model is about male infertility, LD, or KD.

2. The author described a large number of contents related to male infertility in the background, but did not clearly describe the relationship between this animal model and male infertility.

3. All pictures do not describe statistical information in detail, such as statistical methods and the amount included in the analysis.

4. The authors claim that they have successfully developed a LD & KD in MI model, but do not specify the accepted criteria for the identification of this model.
